# Isoxazole derivatives as new nitric oxide elicitors in plants

**DOI:** 10.3762/bjoc.13.65

**Published:** 2017-04-06

**Authors:** Anca Oancea, Emilian Georgescu, Florentina Georgescu, Alina Nicolescu, Elena Iulia Oprita, Catalina Tudora, Lucian Vladulescu, Marius-Constantin Vladulescu, Florin Oancea, Calin Deleanu

**Affiliations:** 1 National Institute of Research and Development for Biological Sciences, Spl. Independentei 296, RO-060031 Bucharest, Romania; 2 Research Center Oltchim, Str. Uzinei 1, RO-240050, Ramnicu Valcea, Romania; 3 Research Dept., Teso Spec SRL, Str. Muncii 53, RO-915200 Fundulea, Calarasi, Romania; 4 Petru Poni Institute of Macromolecular Chemistry, Romanian Academy, Aleea Grigore Ghica Voda 41-A, RO-700487 Iasi, Romania; 5 C. D. Nenitescu Centre of Organic Chemistry, Romanian Academy, Spl. Independentei 202-B, RO-060023 Bucharest, Romania; 6 National Research & Development Institute for Chemistry & Petrochemistry – ICECHIM, Spl. Independentei 202, RO-060021 Bucharest, Romania

**Keywords:** chemical elicitor, 1,3-dipolar cycloaddition, isoxazole, nitric oxide, nitrile oxide, reactive oxygen species

## Abstract

Several 3,5-disubstituted isoxazoles were obtained in good yields by regiospecific 1,3-dipolar cycloaddition reactions between aromatic nitrile oxides, generated in situ from the corresponding hydroxyimidoyl chlorides, with non-symmetrical activated alkynes in the presence of catalytic amounts of copper(I) iodide. Effects of 3,5-disubstituted isoxazoles on nitric oxide and reactive oxygen species generation in *Arabidopsis* tissues was studied using specific diaminofluoresceine dyes as fluorescence indicators.

## Introduction

Isoxazoles are an interesting class of *N*-heterocyclic compounds intensely studied mainly due to their wide range of biological activity [1–2]. Isoxazole compounds show antiviral [3–4], antithrombotic [5–9], analgesic [9], COX-2 inhibitory [10–11], anti-inflamatory [9,11], antinociceptive [12] and anticancer [13] activities. Several isoxazole derivatives have GABA_A_ antagonist [14] and T-type Ca^2+^ channel blocking activities [15]. Commercial drugs featuring an isoxazole moiety include the COX-2 inhibitor Valdecoxib and the β-lactam antibiotics Cloxacillin and Dicloxacillin. An isoxazole derivative, namely 3,5-difluorophenyl-[3-methyl-4-(methylsulfonyl)isoxazol-5-yl]methanone, was recently reported as an inducer of nitric oxide producing elicitor in plants [16–17]. Nitric oxide (NO), which has been demonstrated to be a major gasotransmitter in mammals, is also involved in the orchestration of various plant physiological responses, playing an important role in the regulation of interactions between plant and microorganisms and in plant defense mechanisms against stresses [18–19]. Consequently, there is interest in the biological evaluation of further isoxazole derivatives.

Many synthetic approaches towards the isoxazole core include the reactions of hydroxylamine with aryl-β-diketones [20], α,β-unsaturated carbonyl compounds [21], or α,β-unsaturated nitriles [22], and 1,3-dipolar cycloaddition reactions between alkenes or alkynes and nitrile oxides [23–25].

Nitrile oxides are known as reactive 1,3-dipoles involved in 1,3-dipolar cycloaddition reactions with various dipolarophiles generating five-membered heterocyclic compounds, such as isoxazoles, isoxazolines, oxadiazoles, oxadiazolines, dioxazolidines etc. [23–25]. Intermediate nitrile oxides are usually generated in situ by the oxidative dehydrogenation of aldoximes in the presence of various oxidants [26–29], or by the dehydrohalogenation of hydroxyiminoyl halides promoted by organic or inorganic bases [30–32]. A less used synthetic procedure involves the oxidative dehydration of primary nitro compounds with isocyanates in the presence of tertiary alkylamines [33].

Generally, the cycloaddition reactions of nitrile oxides to alkenes yield isoxazolines or a mixture of isoxalines and isoxazoles. Cycloaddition reactions of nitrile oxides to alkynes yield isoxazoles directly, without a catalyst, but the yields of isoxazole products are quite low because of side reactions and both regioisomers are generally obtained [23–25]. The one-pot 1,3-dipolar cycloaddition reaction of a nitrile oxide, generated in situ from the corresponding hydroxymoyl chloride, with an in situ brominated electron-deficient alkene led to the intermediate bromoisoxazoline from which, by loss of HBr, a 3,5-disubstituted isoxazole derivative is formed as major regioisomer [34]. Based on the copper(I)-catalyzed click reactions of organic azides with terminal acetylenes [35], different copper(I)-catalyzed synthetic procedures towards isoxazole derivatives were developed [36–37].

As part of our continued efforts to develop simple synthetic routes towards bioactive heterocyclic compounds [38–43], we report here the synthesis of several 3,5-isoxazole derivatives, bearing benzo[1,3]dioxole and thiophene scaffolds respectively, as well as their inductor effect on the generation of nitric oxide and reactive oxygen species in plant tissues. The benzo[1,3]dioxole framework is a constituent of some fragrances and flavors [44], and several bioactive compounds with a broad spectrum of applications [45–48]. The thiophene is a core system of a large number of bioactive molecules such as antineoplastic agents [49], non-steroidal anti-inflammatory drugs [50] or compounds with antibacterial activities against several Gram-positive strains [51]. Thus, 3,5-disubstituted isoxazole derivatives were obtained by regioselective 1,3-dipolar cycloaddition reactions of aromatic nitrile oxides to non-symmetrical activated alkyne derivatives in the presence of catalytic amounts of copper(I) iodide.

## Results and Discussion

### Synthesis of 3,5-disubstituted isoxazole derivatives

**Scheme 1:**
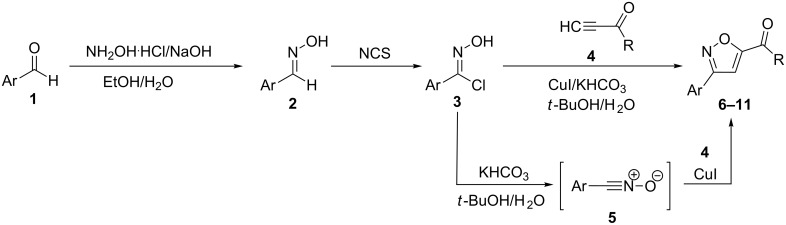
Synthetic route to 3,5-disubstituted isoxazoles.

The regioselective cooper(I)-catalyzed 1,3-dipolar cycloaddition reactions of aromatic nitrile oxides, generated in situ from the corresponding crude imidoyl chlorides, and non-symmetrically activated alkynes led to 3,5-disubstituted isoxazole derivatives. For this, aromatic aldehydes **1** are first converted to the corresponding aldoximes **2**, via reactions with hydroxylamine, and the crude reaction products are transformed to the corresponding imidoyl chlorides **3** which are directly used in the next step without purifications. Catalytic amounts of copper(I) iodide and a base, such as KHCO_3_, are added to an aqueous solution containing the crude imidoyl chlorides **3** and the non-symmetrical activated alkynes **4**. The in situ generated aromatic nitrile oxides **5** undergo an addition to copper(I) acetylides formed in situ to give the 3,5-disubstituted isoxazoles **6–11** as single isomers, in moderate to good yields (Scheme 1).

All reactions between crude imidoyl chlorides **3** and the non-symmetrical activated alkynes **4** are carried out in aqueous solutions at room temperature. The final 3,5-disubstituted isoxazoles are easily separated by filtration, washed with water and recristallysed. The synthesized 3,5-disubstituted isoxazoles are presented in Table 1.

**Table 1:** Reported 3,5-disubstituted isoxazoles.

Compound	Ar	R	mp (º)	Yield (%)

**6**	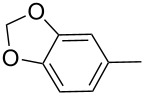	Me	159–160; 141–142 [51]	81
**7**		OEt	100–101.5	70
**8**		Ph	135–137	78

**9**	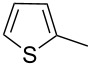	Me	113–115	73
**10**		OEt	78–80	67
**11**		Ph	90–91	74

3,5-Disubstituted isoxazoles **6**, **7**, **9** and **10** (Table 1) have been previously prepared by different synthetic procedures, but compounds **6** [52], **7** [49] and **10** [53] have been only partly characterized, while for compound **9** [54] no characterization data have been reported.

3,5-Disubstituted isoxazole structures were unambiguously assigned on the basis of chemical and spectral analysis (IR, ^1^H, ^13^C and ^15^N NMR spectra). NMR spectra clearly indicated the presence of only one regioisomer for all synthesized 3,5-disubstituted isoxazoles. Signals in ^1^H and ^13^C NMR spectra were fully assigned based on H,C-HSQC and H,C-HMBC experiments. The 3,5-disubstitution was also experimentally proven by NOE experiments which indicated through space interaction between the H-4 proton and the protons from both 3- and 5- substituents.

### Biological activity

We investigated the inductor effects of 3,5-disubstituted isoxazoles **6–11** on NO and reactive oxygen species (ROS) production in plant tissues. Usually, NO and ROS, such as O_2_^−^, OH· and H_2_O_2_, together are required to induce the activation of various defense-related enzymes in plants [55]. Plant cells contain oxygen radical detoxifying enzymes and nonenzymatic antioxidants which have an essential role in protection of plant cells from oxidative damages at the sites of ROS and NO generation [56–57]. Measuring the ROS and NO levels in plant tissues is often difficult due to high reactivity and extremely short physiological half-life of these free radicals [58–59].

In this work, generation of both NO and ROS was proven by fluorescence microscopy using specific fluorescence indicators that help to exactly define the sites of generation. We used *Arabidopsis thaliana* wild type seeds, cultivated for six weeks in laboratory in Arasystem [60]. *Arabidopsis thaliana* was selected as model organisms for NO inductors because this is the flowering plant with the largest amount of knowledge on cellular and molecular biology and it has a relatively short life cycle. The *Arabidopsis* leaves were sprayed with fine suspensions of isoxazole inductors **6–11** at two different concentrations (10 μg/mL and 50 μg/mL, respectively) and collected after 24 h. Collected leaves were washed with distilled water and incubated with the specific fluorescence indicator for histochemical analysis of ROS and NO by fluorescence microscopy. *Arabidopsis* leaves untreated with inductor suspensions have been used as negative controls. As positive control, we used chitosan (β-1,4 linked glucosamine) with average molecular weight, a fungal elicitor with known effect as NO and ROS inductor on *A. thaliana* [61].

Intracellular ROS was visualized using 2’,7’-dichlorodihydrofluorescein diacetate (H_2_DCF DA) as fluorescence indicator. The method is based on the oxidation of the non-fluorescent probe of 2’,7’-dichlorodihydrofluorescein diacetate to the highly fluorescent 2’,7’-dichlorofluorescein diacetate [62–63].

Intracellular NO was visualized using 4-amino-5-methylamino-2’,7’-dichlorodihydrofluorescein diacetate (DAF-FM diacetate), a non-fluorescent compound, which reacts with NO to form a fluorescent benzotriazole and does not react with any ROS [64–67].

Fluorescence microscopy images of all 3,5-disubstituted isoxazoles*-*treated *Arabidopsis* leaves showed a pronounced presence of ROS at both concentrations of inductors (10 μg/mL and 50 μg/mL). Strong fluorescence densities were observed especially at higher concentration (50 μg/mL) of 3,5-disubstituted isoxazoles. Intensities of fluorescence revealed differences between tested compounds. These compounds can be listed in the increasing order of ROS generation efficacy as follows: **11** > **10** > **9** > **7** > **8** > **6** (Figure S2 in Supporting Information File 1).

Similarly, images of fluorescence microscopy revealed the presence of NO in all 3,5-disubstituted isoxazoles*-*treated *Arabidopsis* leaves, at both concentrations (10 μg/mL and 50 μg/mL), especially at higher concentration of compounds (50 μg/mL). The NO releasing capacity of newly synthesized 3,5-disubstituted isoxazoles followed the series: **9** > **11** > **10** > **8** > **7** = **6** (Figure S3 in Supporting Information File 1).

Fluorescence data indicate that the 3,5-disubstituted isoxazoles **6–11**, particularly **9–11**, are involved in NO and ROS production in *Arabidopsis* treated leaves.

## Conclusion

Several 3,5-disubstituted isoxazoles were obtained by the convenient, regiospecific 1,3-dipolar cycloaddition reactions of aromatic nitrile oxides, generated in situ from the crude imidoyl chlorides, with non-symmetrical activated alkynes in the presence of catalytic amounts of copper(I) iodide. The effect of 3,5-disubstituted isoxazoles in generation of ROS and NO in plant tissues was investigated by fluorescent microscopy. The obtained data indicate that some of these compounds are chemical elicitors that induce NO and ROS generation in plant tissues and could activate various defense mechanisms in plants. Further research is in progress to assess the in planta mechanism of NO generation by these compounds.

## Supporting Information

File 1 Experimental procedures, characterization data, IR, ^1^H, ^13^C and ^15^N NMR data for all new compounds and fluorescence microscopy images of NO and ROS generation for all 3,5-disubstituted isoxazoles*-*treated *Arabidopsis* leaves.
