# Supplementary material for: Isoxazole derivatives as new nitric oxide elicitors in plants
Source: Beilstein J Org Chem. 2017 Apr 6;13:659–64. doi: 10.3762/bjoc.13.65 (PMC5389174; doi:10.3762/bjoc.13.65)
Supplement: File 1 — Experimental procedures, characterization data, IR, 1H, 13C and 15N NMR data for all new compounds and fluorescence microscopy images of NO and ROS generation for all 3,5-disubstituted isoxazoles-treated Arabidopsis leaves. [file Beilstein_J_Org_Chem-13-659-s001.pdf]

Supporting Information

for

Isoxazole derivatives as new nitric oxide elicitors in plants

Anca Oancea[1], Emilian Georgescu[2], Florentina Georgescu[3], Alina Nicolescu[4,5], Elena Iulia Oprita[1], Catalina Tudora[1], Lucian Vladulescu[3], Marius-Constantin Vladulescu[3], Florin Oancea[6] and Calin Deleanu*[4,5]

Address: [1]National Institute of Research and Development for Biological Sciences, Spl. Independentei 296, RO-060031 Bucharest, Romania, [2]Research Center Oltchim, Str. Uzinei 1, RO-240050, Ramnicu Valcea, Romania, [3]Research Dept., Teso Spec SRL, Str. Muncii 53, RO-915200 Fundulea, Calarasi, Romania, [4]“Petru Poni” Institute of Macromolecular Chemistry, Romanian Academy, Aleea Grigore Ghica Voda 41-A, RO-700487 Iasi, Romania, [5]“C. D. Nenitescu” Centre of Organic Chemistry, Romanian Academy, Spl. Independentei 202-B, RO-060023 Bucharest, Romania [6]National Research & Development Institute for Chemistry & Petrochemistry – ICECHIM, Spl. Independentei 202, RO-060021 Bucharest, Romania.

Email: Calin Deleanu* - calin.deleanu@yahoo.com

*Corresponding author

Experimental procedures, characterization data, IR, ^{1}H, ^{13}C and ^{15}N NMR data for all new compounds and fluorescence microscopy images of NO and ROS generation for all 3,5-disubstituted isoxazoles-treated *Arabidopsis* leaves

Table of contents

General experimental details.

General procedure and spectral data for compounds **6-11**

General experimental details for fluorescence measurements

General test procedure and fluorescence microscopy images of NO and ROS in *Arabidopsis* tissues.

References

General experimental details

Melting points were measured on Boëtius hot plate microscope and are uncorrected. IR spectra were recorded on a Nicolet Impact 410 spectrometer, in KBr pellets. The NMR spectra have been recorded on a Bruker Avance III 400 instrument operating at 400.1, 100.6 MHz and 40.5 MHz for ^{1}H, ^{13}C, and ^{15}N nuclei, respectively. Samples were transferred in 5 mm Wilmad 507 NMR tubes and recorded with a 5 mm multinuclear inverse detection z-gradient probe. Chemical shifts are reported in δ units (ppm) and were referenced to internal TMS for ^{1}H chemical shifts, to the internal deuterated solvent for ^{13}C chemical shifts ($CDCl_3$ referenced at 77.0 ppm), and referenced to liquid ammonia (0.0 ppm) using nitromethane (380.2 ppm) as external standard for ^{15}N chemical shifts. Unambiguous 1D NMR signal assignments were made based on 2D NMR homo- and heteronuclear correlations. H,H-COSY, H,H-NOESY, H,C-HSQC and H,C-HMBC experiments were recorded using standard pulse sequences in the version with z-gradients, as delivered by Bruker with the TopSpin 2.1 PL6 spectrometer control and processing software. The ^{15}N chemical shifts were obtained as projections from the 2D indirectly detected H,N-HMBC spectra, employing a standard pulse sequence in the version with z-gradients as

delivered by Bruker (TopSpin 2.1 PL6). Elemental analyses for C, H and N were obtained using a COSTECH Instruments EAS32. Satisfactory microanalyses for all new compounds were obtained.

Intermediary imidoyl chlorides were prepared according to reported methods [1-3]. Aromatic aldehides, ethyl propiolate, 3-butyn-2-one, 1-phenylpropynone, copper(I) iodide and solvents were purchased from Sigma Aldrich and used further without purification.

General procedure for synthesis of 3,5-disubstituted isoxazoles

To a solution of crude imidoyl chlorides (10 mmol) in 60 mL of 1:1 *tert*-BuOH:H_2O were added under stirring a non-symmetrical activated alkyne (10 mmol) and copper(I) iodide (15 mg). Then, $KHCO_3$ (4.33 g, 43.25 mmol) was added in small portions and the reaction mixture was stirred at room temperature for one hour. The solid was filtered off, washed with water and recrystallized.

1-(3-(Benzo[1,3]dioxol-5-yl)isoxazol-5-yl)ethanone (6) was obtained as pale yellow crystals, yield 81% (1.873 g), mp 159-160 ºC (from $CHCl_3$/MeOH), IR (ν_{max}, cm^{-1}): 3116, 2913, 1689, 1577, 1510, 1464, 1439, 1362, 1290, 1243, 1204, 1120, 1033. ^1H NMR (400.1 MHz, $CDCl_3$) δ (ppm): 2.63 (3H, s, CH_3), 6.03 (2H, s, CH_2), 6.89 (1H, d, 8.0 Hz, H-5'), 7.10 (1H, s, H-4), 7.28 (1H, dd, 8.04, 1.70 Hz, H-6'), 7.33 (1H, d, 1.6 Hz, H-2'). ^{13}C NMR (100.6 MHz, $CDCl_3$) δ (ppm): 27.2 (CH_3), 101.6 (CH_2), 104.9 (C-4), 106.9 (C-2'), 108.7 (C-5'), 121.4 (C-6'), 121.8 (C-1'), 148.4 (C-3'), 149.6 (C-4'), 162.8 (C-3), 166.9 (C-5), 186.9 (CO). ^{15}N NMR (40.6 MHz, $CDCl_3$) δ (ppm): 376.6. Anal. Calcd for $C_{12}H_9NO_4$ (231.20): C, 62.34 %; H, 3.92 %; N, 6.06 %. Found: C, 62.29 %; H, 4.02 %; N, 6.10 %.

3-(Benzo[1,3]dioxol-5-yl)isoxazole-5-carboxylic acid ethyl ester (7) was obtained as white solid, yield 70% (1.83 g), mp 100-101.5ºC (from $CHCl_3$/MeOH), IR (ν, cm^{-1}): 3131, 2906, 1740, 1588, 1468, 1449, 1309, 1216, 1161, 1109, 1037. ^1H NMR (400.1

MHz, $CDCl_3$) δ (ppm): 1.47 (3H, t, 7.1 Hz, CH_3), 4.50 (2H, q, 7.1 Hz, CH_2), 6.08 (2H, s, CH_2), 6.93 (1H, d, 8.1 Hz, H-5'), 7.20 (1H, s, H-4), 7.33 (1H, dd, 8.08, 1.30 Hz, H-6'), 7.38 (1H, d, 1.3 Hz, H-2'). ^{13}C NMR (100.6 MHz, $CDCl_3$) δ (ppm): 14.1 (CH_3), 62.3 (CH_2), 101.6 (CH_2), 106.9 (C-2'), 107.2 (C-4), 108.7 (C-5'), 121.4 (C-6'), 121.9 (C-1'), 148.4 (C-3'), 149.6 (C-4'), 156.9 (C-5), 160.8 (C-3), 162.5 (COO). ^{15}N NMR (40.6 MHz, $CDCl_3$) δ (ppm): 377.5. Anal. Calcd for $C_{13}H_{11}NO_5$ (261.23): C, 59.77 %; H, 4.24 %; N, 5.36 %. Found: C, 59.70 %; H, 4.29 %; N, 5.40 %.

(3-(Benzo[1,3]dioxol-5-yl)isoxazol-5-yl)phenylmethanone (8) was obtained as pale yellow, yield 78% (2.29 g), mp 135-137ºC (from $CHCl_3$/MeOH), IR (v, cm^{-1}): 3160, 2921, 1651, 1596, 1514, 1459, 1420, 1254, 1232, 1106, 1032. ^{1}H NMR (400.1 MHz, $CDCl_3$) δ (ppm): 6.05 (2H, s, CH_2), 6.91 (1H, d, 8.1 Hz, H-5'), 7.26 (1H, s, H-4), 7.34 (1H, dd, 8.08, 1.08 Hz, H-6'), 7.39 (1H, d, 1.04 Hz, H-2'), 7.56 (2H, t, 7.7 Hz, H-3"), 7.68 (1H, t, 7.4 Hz, H-4"), 8.18 (2H, d, 7.6 Hz, H-2"). ^{13}C NMR (100.6 MHz, $CDCl_3$) δ (ppm): 101.6 (CH_2), 107.0 (C-2'), 108.0 (C-4), 108.8 (C-5'), 121.5 (C-6'), 121.9 (C-1'), 128.8 (C-3"), 130.0 (C-2"), 134.1 (C-4"), 135.5 (C-1"), 148.4 (C-3'), 149.6 (C-4'), 162.3 (C-3), 167.3 (C-5), 181.4 (CO). ^{15}N NMR (40.6 MHz, $CDCl_3$) δ (ppm): 376.3. Anal. Calcd for $C_{17}H_{11}NO_4$ (293.27): C, 69.62 %; H, 3.78 %; N, 4.78 %. Found: C, 69.71 %; H, 3.82 %; N, 4.60 %.

1-(3-(Thiophen-2-yl)isoxazol-5-yl)ethanone (9) was obtained as beige crystals, yield 73% (1.41 g), mp 113-115ºC (from MeOH), IR (v_{max}, cm^{-1}): 3109, 3094, 1695, 1584, 1539, 1460, 1393, 1365, 1290, 1205, 1097, 1018. ^{1}H NMR (400.1 MHz, $CDCl_3$) δ (ppm): 2.68 (3H, s, CH_3), 7.15 (1H, s, H-4), 7.18 (1H, t, 3.9 Hz, H-3'), 7.51 (1H, d, 5 Hz, H-4'), 7.54 (1H, d, 3.2 Hz, H-2'). ^{13}C NMR (100.6 MHz, $CDCl_3$) δ (ppm): 27.2 (CH_3), 105.0 (C-4), 127.9 (C-3'), 128.2 (C-2'), 128.4 (C-4'), 129.5 (C-1'), 158.4 (C-3), 166.9 (C-5), 186.8 (CO). ^{15}N NMR (40.6 MHz, $CDCl_3$) δ (ppm): 374.2. Anal. Calcd for

$C_9H_7NO_2S$ (193.22): C, 55.94 %; H, 3.65 %; N, 7,25 %. Found: C, 56.0 %; H, 3.68 %; N 7.20 %.

3-(Thiophen-2-yl)isoxazole-5-carboxylic acid ethyl ester (10) was obtained as beige crystals, yield 67% (1.50 g), mp 78-80ºC (from MeOH), IR (ν, cm^{-1}): 3129, 3112, 2979, 1728, 1546, 1463, 1366, 1289, 1236, 1131, 1110, 1015. ^1H NMR (400.1 MHz, $CDCl_3$) δ (ppm): 1.47 (3H, t, 7.1 Hz, CH_3), 4.49 (2H, q, 7.1 Hz, CH_2), 7.17 (1H, t, 4.1 Hz, H-3’), 7.20 (1H, s, H-4), 7.50 (1H, d, 4.7 Hz, H-4’), 7.54 (1H, d, 3.1 Hz, H-2’). ^{13}C NMR (100.6 MHz, $CDCl_3$) δ (ppm): 14.1 (CH_3), 62.4 (CH_2), 107.2 (C-4), 127.8 (C-3’), 128.1 (C-2’), 128.4 (C-4’), 129.6 (C-1’), 156.6 (C-3), 158.2 (C-5), 160.8 (COO). ^{15}N NMR (40.6 MHz, $CDCl_3$) δ (ppm): 375.7. Anal. Calcd for $C_{10}H_9NO_3S$ (223.25): C, 53.80 %; H, 4.06 %; N, 6.27 %. Found: C, 53.77 %; H, 4.0 %; N, 6.31 %.

Phenyl-(3-(thiophen-2-yl)isoxazol-5-yl)methanone (11) was obtained as a beige solid, yield 74% (1.89 g), mp 90-91ºC (from MeOH), IR (ν, cm^{-1}): 1652, 1573, 1537, 1456, 1392, 1261, 1233, 1184, 1025. ^1H NMR (400.1 MHz, $CDCl_3$) δ (ppm): 7.18 (1H, t, 4.0 Hz, H-3’), 7.28 (1H, s, H-4), 7.51 (1H, d, 4.9 Hz, H-4’), 7.58 (1H, d, 5.2 Hz, H-2’), 7.58 (2H, t, 8.2 Hz, H-3’’), 7.71 (1H, t, 7.4 Hz, H-4”), 8.20 (2H, d, 7.5 Hz, H-2”). ^{13}C NMR (100.6 MHz, $CDCl_3$, δ (ppm)): 108.0 (C-4), 127.9 (C-3’), 128.3 (C-2’), 128.4 (C-4’), 128.8 (C-3”), 129.5 (C-1’), 130.0 (C-2”), 134.2 (C-4”), 135.4 (C-1”), 157.9 (C-3), 167.3 (C-5), 181.1 (CO). ^{15}N NMR (40.6 MHz, $CDCl_3$) δ (ppm): 374.4. Anal. Calcd for $C_{14}H_9NO_2S$ (255.29): C, 65.87 %; H, 3.55 %; N, 5.49 %. Found: C, 65.93 %; H, 3.64 %; N 5.42 %.

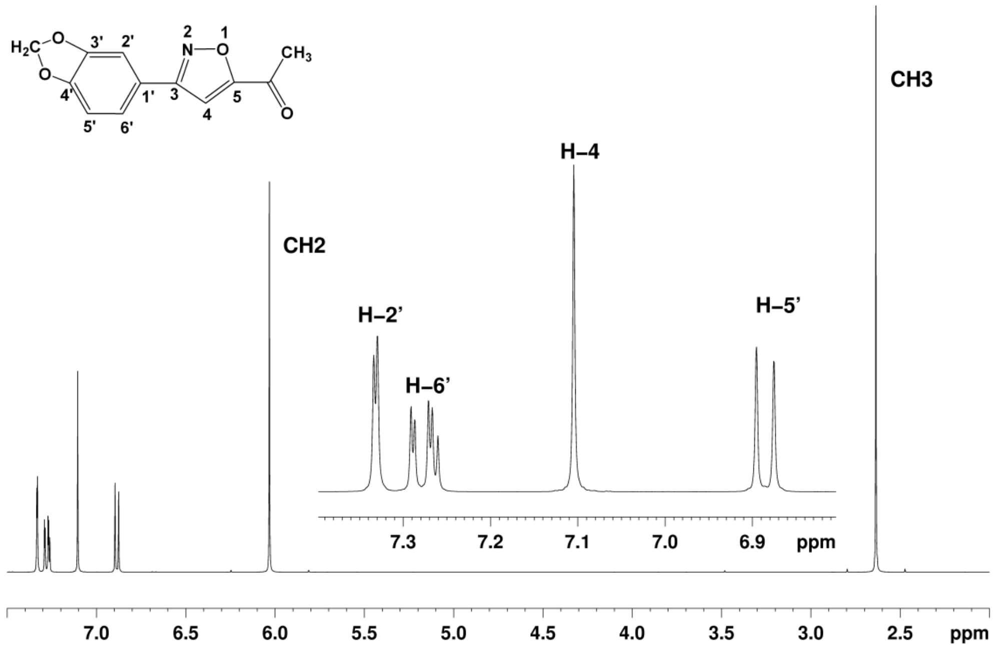

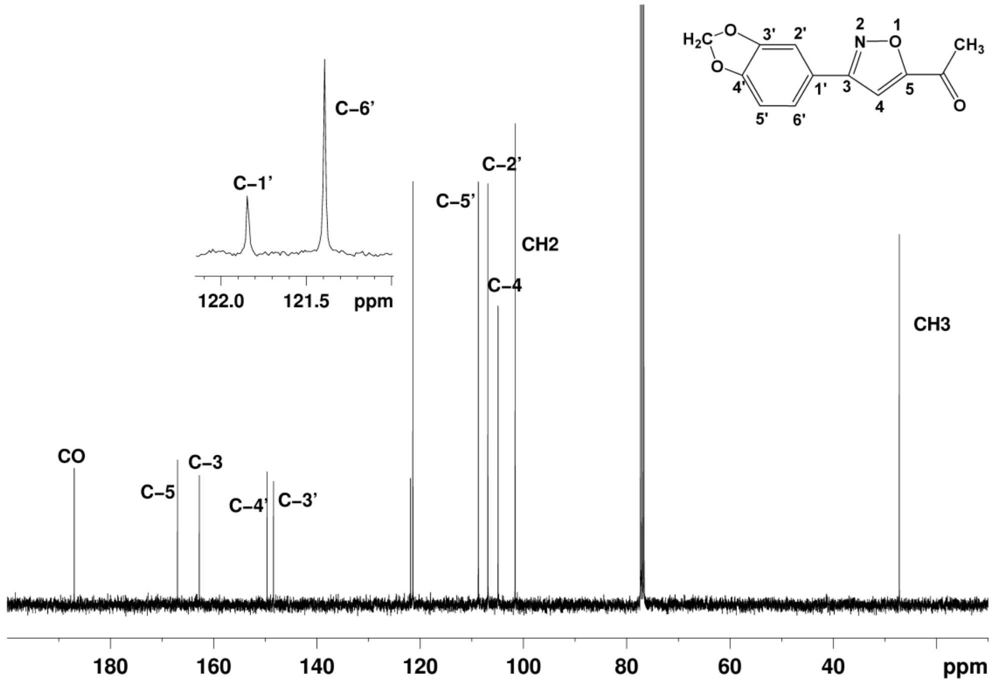

Figure S1. ^{1}H (top) and ^{13}C (bottom) as examples of NMR spectra corresponding to compound **6**, recorded in $CDCl_3$.

General experimental details for fluorescence measurements

Fluorescence measurements were recorded on an AXIO - OBSERVER D1, Zeiss, equipped with a video digital camera AxioCam MRc using an AxioVision Rel.4.6 software.

Fluorescence indicator H_2DCF DA (2',7'-dichlorodihydrofluorescein diacetate) was purchased from Invitrogen Molecular Probes. DAF-FM DA (4-amino-5-methylamino-2',7'-dichlorodihydrofluorescein diacetate), chitosan with average molecular weight and TWEEN 20, a nonionic detergent, were purchased from Sigma Aldrich.

General test procedure and fluorescence microscopy images of NO and ROS in *Arabidopsis* tissues.

The experiments have been performed on *Arabidopsis thaliana* plants, cultivated in a laboratory in Arasystem [4]. We used *Arabidopsis thaliana* wild type seeds (provided by *Lehke Seeds Texas, USA*) that have been seeded in sterilized soil and cultivated for six weeks in a special growth room, at 21–23 °C, 70% humidity, light intensity 150 µmol/m^2 and a photoperiod of 14 h/10 h.

Each 3,5-disubstituted isoxazole compound, 0.5 mg, and 2.5 mg respectively, dissolved in 1 mL etanol was mixed with 0.25 g Tween 20 and demineralized water to prepare 50 mL of each test suspension. Test suspensions were kept in spraying glass bottle, in the dark, at room temperature. The *Arabidopsis* leaves were sprayed with the inductor suspensions (at a rate of 1 mL/plant) and collected after 24 h. As positive control we used plant treated with chitosan solution, 50 µg/mL, in 0.5% acetic acid solution, buffered to pH 5.6 with NaOH 1 M.

Histochemical analysis

Intracellular ROS was visualized by using H_2DCF DA as fluorescence indicator. The collected *Arabidopsis* leaves were washed with distilled water and incubated with 2.5 µM H_2DCFA solution (10 mM in DMSO), for 30 minute in the dark, at room temperature. Then the leave fragments were washed twice with distilled water and the H_2DCF DA – mediated fluorescence was detected (emission/excitation: 488/525 nm). *Arabidopsis* leaves untreated with inductor suspensions were used as negative control. ROS generation in *Arabidopsis* leaves after 24 hour from treatment with inductor suspensions, of 3,5-disubstituted izoxazole or chitosan, at concentrations of 50 µg/mL, is presented in Figure S2.

Intracellular NO was visualized by using DAF-FM DA fluorescence indicator (Figure S2). The collected *Arabidopsis* leaves were washed with distilled water and incubated with 10 µM DAF-FM diacetate (5 mM in DMSO), for 15 min, in the dark, at room temperature. Then the leave fragments were washed twice with phosphate buffer saline (PBS) at pH 7.4 and the fluorescence of the reaction product of DAF-FM DA with NO was captured (emission/excitation: 488/525 nm). *Arabidopsis* leaves untreated with inductor suspensions were used as negative controls. NO generation in *Arabidopsis* leaves after 24 hour from treatment with inductor suspensions of 3,5-disubstituted izoxazole or chitosan, at concentrations of 50 µg/mL, is presented in Figure S3.

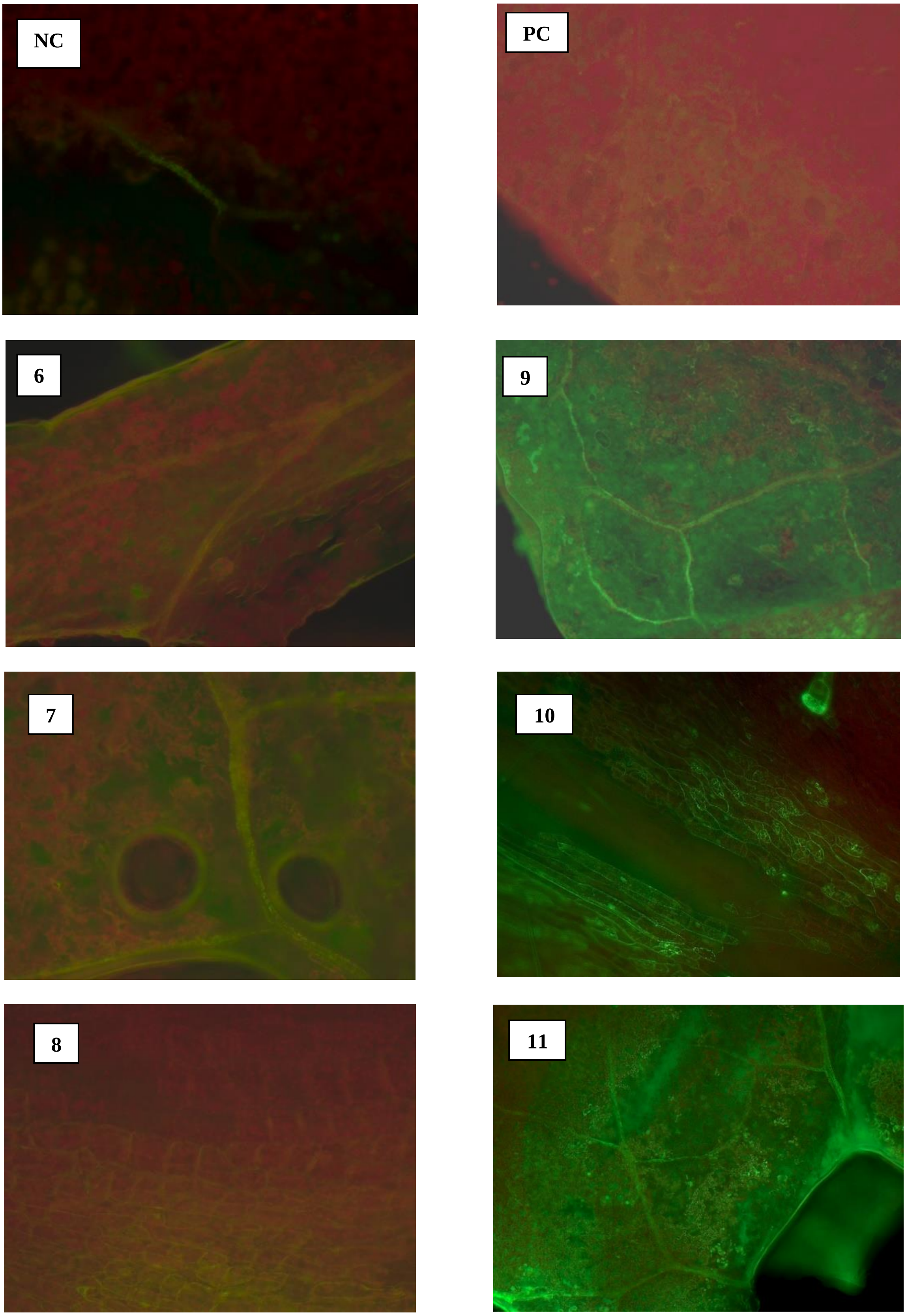

Figure S2. ROS generation in *Arabidopsis* leaves after 24 hour from treatment with inductor suspensions (NC = negative control; PC = positive control, chitosan)

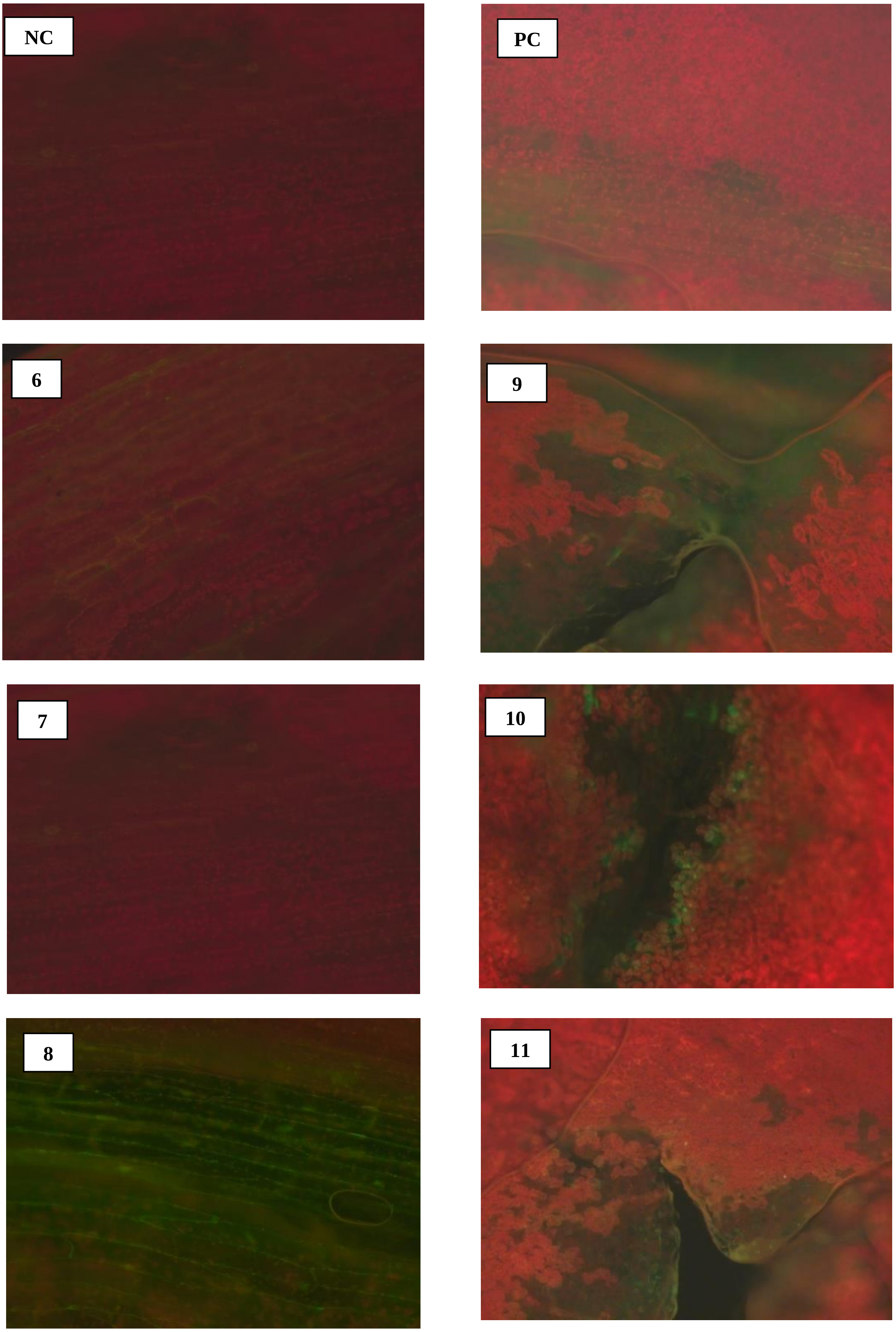

Figure S3. NO generation in *Arabidopsis* leaves after 24 hour from treatment with inductor suspensions (NC = negative control; PC = positive control, chitosan)

References

[1] Grundmann, C.; Richter, R. *J. Org. Chem*. **1968**, *33*, 476-478.

[2] Liu, K.-C.; Shelton, B. R.; Howe, R.K. *J. Org. Chem*. **1980**, *45*, 3916-3918.

[3] Hallig, K.; Torssell, K. B. G.; Hazell, R. G. *Acta Chem. Scand*. **1991**, *45*, 736-741.

[4] D. Weigel, J. Glazebrook (Eds.), Arabidopsis: a laboratory manual, Cold Spring Harbor Laboratory Press, New York, 2002, pp. 143-170.
